# Here today, gone tomorrow? Managerial perceptions of opportunities and challenges with temporary employment in eldercare

**DOI:** 10.1186/s12913-026-15246-y

**Published:** 2026-07-29

**Authors:** Tomas Lindmark, David Hallman, Gunnar Bergström, Annika Strömberg

**Affiliations:** 1 Department of Social Work, Uppsala University, Uppsala, Sweden; 2 Faculty of Health and Occupational Studies, Department of Social Work, Criminology and Public Health, University of Gävle, Gävle, Sweden; 3 Faculty of Health and Occupational Studies, Department of Occupational Health, Psychology and Sports Sciences, University of Gävle, Gävle, Sweden; 4 Unit of Intervention and Implementation Research for Worker Health, Institute of Environmental Medicine, Karolinska Institutet, Stockholm, Sweden; 5 Faculty of Education and Business Studies, Department of Business and Economics Studies, University of Gävle, Gävle, Sweden

**Keywords:** Flexible employment, Home care, Precarious work, Residential care home, Staffing shortages, Work environment, Zero-hour contracts

## Abstract

**Background:**

Temporary employment is widely used in eldercare to address staffing shortages, but heavy reliance on temporary workers may compromise continuity of care, staff workload, and quality of care. Managers are key to how staffing is organised and handled in daily practice. This study examined how managers at different organisational levels perceived the opportunities and challenges of temporary employment in municipal eldercare.

**Methods:**

This qualitative study was based on semi-structured interviews with 16 managers across four organisational levels in Swedish municipal eldercare, including residential care homes, home care services, and higher-level management. The interview guide focused on managers’ experiences of temporary employment, staffing practices, care continuity, work environment, and perceived effects on care quality. Data were analysed using qualitative content analysis.

**Results:**

Three themes were identified. *Balancing staffing on a tightrope* describes managers’ struggle to ensure continuity in a system that relies on short-term solutions and often prioritising shift coverage over competence. *Leading with tied hands* captures managers’ limited control over recruitment and workforce planning, which reinforced reliance on temporary employment. *From rigidity to flexibility* highlights managers’ suggestions for improving staffing stability. Managers across levels identified similar challenges, but their priorities differed. First-line managers focused on daily staffing, staff well-being, and quality of care, while higher-level managers emphasised long-term planning. Temporary workers helped cover staffing gaps, but also increased permanent employees’ workloads and complicated workforce planning. Managers expressed frustration with centralised staffing decisions that prioritised immediate coverage over long-term stability. Temporary workers were often treated as interchangeable rather than integrated team members, reflecting systemic problems in staffing practices.

**Conclusion:**

Temporary workers are essential for keeping eldercare services running, but over-reliance on temporary employment may reinforce reactive staffing practices, increase pressure on permanent staff, and weaken continuity. Managers suggested better onboarding, greater local hiring control, and dedicated staffing pools. Addressing these issues requires both immediate measures to integrate temporary workers into regular teams and longer-term changes in workforce planning, staffing levels, and local managerial influence.

**Supplementary Information:**

The online version contains supplementary material available at 10.1186/s12913-026-15246-y.

## Background

For decades, eldercare worldwide has faced persistent challenges, including a global shortage of qualified staff, low wages, high sick leave and staff turnover, which undermine both care quality and workforce sustainability [1–3]. These systemic issues are intensified by underfunding [4], leaving organisations reliant on temporary workers to fill gaps [5–7]. Temporary workers - commonly employed on casual, fixed-term, or zero-hours contracts - constitute a considerable portion of the eldercare workforce. In Sweden, temporary workers are often hired on an on-call basis, meaning they work only when the employer needs them and are paid for the hours they are called in. They constitute 20–25% of the workforce in eldercare, with municipal variation ranging from 8% to 40%. The proportion of temporary workers in eldercare is notably higher than the national average for temporary employment across other sectors [8–11].

Staff continuity is important for the quality of care, as it allows care workers to build trust with residents, respond to individual needs, and detect changes in health and behaviour over time [12, 13]. Temporary workers play a key role in ensuring that eldercare services can operate around the clock. At the same time, they often receive less training, have limited access to supervision and team support, and may face job insecurity, which can make it more difficult to maintain consistent care standards [5, 14]. This makes introductory practices important, as adequate orientation in residential long-term care has been associated with job satisfaction [15]. A high degree of staff rotation can make it harder to maintain shared values and routines over time [16]. Since temporary workers are not always familiar with residents or work processes, this may contribute to fragmented care. Some temporary workers describe their situation as marked by uncertainty, limited control, and the need always to be available, which can make daily life difficult and contribute to feelings of vulnerability [17].

These conditions also influence the work environment for permanent staff, who are often expected to support temporary colleagues alongside their own responsibilities, thereby increasing workload and stress [18, 19]. This is consistent with studies in nursing homes and home care linking staff strain, job satisfaction, and the overall work environment to modifiable organisational conditions, including leadership, job demands, control at work, and available resources [20, 21]. In settings that already face recruitment and retention challenges, frequent staff changes may reduce team stability and lower organisational commitment [6]. Temporary workers in eldercare report lower job demands than permanent staff, particularly in terms of physical strain and workload. This may lead to permanent employees taking on more demanding or complex tasks [8], which may reflect differences in responsibility levels rather than workload distribution. The lack of stability and support may also adversely affect the well-being of temporary workers, contributing to stress and reduced motivation [7, 16, 22]. Despite the growing reliance on temporary workers, research on their working environment remains scarce [14]. Given this extensive use, temporary employment is also a question of how staffing is organised and managed. Since decisions about temporary workers are made across several managerial levels, managers’ organisational positions may influence how temporary employment is understood in relation to staff continuity and quality of care.

The organisation and management of eldercare are important to workforce stability, work environment, and service delivery. Across eldercare systems, managers must balance cost control, staffing needs, care quality, and support for staff. This requires organisational conditions that enable clear workplace roles and sufficient resources [23–25]. In Sweden and other Nordic countries, this balancing act has been influenced by municipal governance, budget cuts, marketisation, and New Public Management ideals, including cost-efficiency, performance monitoring, and tighter resource control [26–28]. This makes Swedish municipal eldercare a useful case for examining how common staffing challenges take form within a publicly funded, municipally governed, and regulated welfare system.

Within this system, responsibility for continuity and quality of care is distributed across several managerial levels. Multi-tiered management structures influence how managerial roles are carried out and perceived. First-line managers are primarily responsible for daily operations and staff supervision, but often have limited influence over recruitment and strategic planning [25]. In contrast, middle managers operate between strategic and operational levels, translating broader HR policies into local practices [29]. Research highlights that managerial tasks, required skills, and time horizons vary between levels. First-line managers tend to implement existing routines within their unit, while middle and higher-level managers coordinate across functions and focus on strategy and long-term planning [30, 31]. However, unclear goals, conflicting demands, and limited autonomy at various levels can lead to inefficiencies, workplace dilemmas, and increased turnover intentions among staff [4, 24, 32]. For managers, temporary employment is also a recurring part of daily staffing, as they must cover shifts while maintaining continuity.

Given these ongoing challenges, it is important to understand how managers at different organisational levels perceive and manage reliance on temporary employment in eldercare. Previous research shows that managerial work varies by position in the organisational hierarchy [25, 29–31]. First-line managers are closer to daily staffing problems and care delivery, whereas higher-level managers are more involved in resource allocation and long-term workforce planning. A level-based perspective can therefore help show both shared challenges and differences between managerial levels.

Thus, this study aims to explore how managers at different organisational levels in Swedish municipal eldercare perceive the opportunities and challenges of temporary employment. Particular attention is paid to contextual and organisational conditions, staffing practices, working conditions, and care quality, as well as how these perceptions vary by organisational position.

In this study, staff working conditions refer to how managers describe the everyday work situation of care workers in relation to temporary employment. The study also considers how managers describe their own work in handling temporary staffing, particularly at the first-line level. Care quality refers to managers’ perceptions of how temporary staffing may affect continuity and the care provided to older adults.

## Methods

### Study design

This study used an inductive qualitative design to explore managers’ perceptions of temporary employment in municipal eldercare. The analysis aimed to stay close to managers’ descriptions while identifying broader patterns across organisational levels. The study is part of the larger research programme *Flexible Work: Opportunity and Challenge (FLOC)*, which examines non-standard employment and flexible work arrangements in Sweden [33].

### Setting

In Sweden, eldercare follows a “social care model” that places greater emphasis on social support than on medical interventions. Residential care homes primarily accommodate older adults with significant care needs who are no longer able to live independently [28]. Home care is the most common eldercare service and supports older adults living at home with personal care, household tasks, and social interactions, as needed [11]. Swedish eldercare is mainly publicly funded and organised at the municipal level. Municipalities are responsible for providing eldercare services, but national legislation, labour regulations, local budgets, and municipal staffing systems influence how services are organised locally. This means that some of the rules and resources affecting daily staffing are set outside the individual unit [26–28].

The workforce in both residential care homes and home care mainly consists of assistant nurses, who typically have formal training, and care aides, who often have less formal education. Temporary workers, however, do not always fall into these categories and may lack similar qualifications. Many temporary workers are employed on an on-call basis, hired per shift without guaranteed working hours, and often lack specific care education or training [11]. In municipal eldercare, temporary workers are commonly used to cover short-term staffing needs, such as sickness absence, holidays, vacancies, and fluctuations in daily staffing requirements. In this study, the term temporary workers primarily refers to workers employed directly by the municipal eldercare organisation on an on-call or short-term basis. This differs from agency staffing, where workers are employed by a third-party staffing agency and brought in to cover staffing needs. Agency staffing is also used in Swedish eldercare and may include formally trained staff, such as assistant nurses or licensed professionals. However, agency staffing was not the focus of the interviews and is therefore not analysed as a separate employment form.

### Sample

**Fig. 1:**
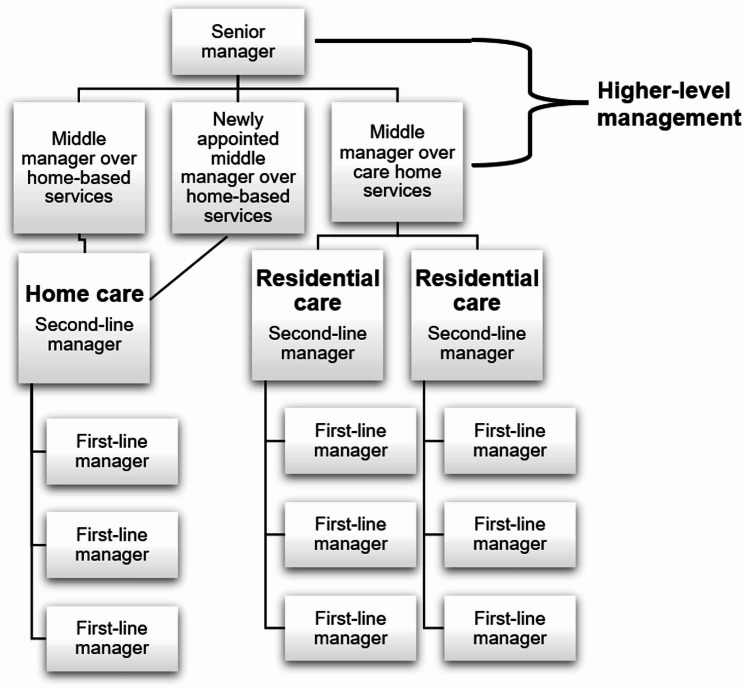
The organisational structure of the included managers in relation to eldercare

The participants were recruited from one Swedish municipality. The sample consisted of 16 managers at four different hierarchical levels within eldercare services. These levels included one senior manager, three middle managers, three second-line managers, and nine first-line managers. Among the first-line managers, six were primarily responsible for residential care homes, and three for home care services. Two of these managers also had responsibilities in both care settings. Participants at the middle and senior levels (levels 3 and 4) were recruited through direct invitations to all eligible managers at the higher levels, and those who agreed to participate were included. To ensure confidentiality and given that only one participant represented level 4 (senior management), levels 3 and 4 were merged into a single category (higher-level management) in the analysis. All second-line managers who were responsible for eldercare facilities chose to participate in the study. To recruit first-line managers (level 1), second-line managers (level 2) were asked to select 3–4 first-line managers each, ensuring a mix of individuals with both long and short experience and with units located in both rural and urban areas. The selection aimed for variation in gender and service type. Residential care homes were slightly overrepresented in the sample due to their larger organisational size, which often includes two first-line managers per care home. Figure 1 shows the organisational structure from the first line to senior management. However, it does not fully reflect the scope of responsibilities. Many managers (levels 2–4) oversaw not only eldercare but also services such as disability care. They also managed more staff than shown. The senior manager supervised about four middle managers; each middle manager oversaw 4–5 second-line managers, and each second-line manager was responsible for 9–13 first-line managers.

Of the 16 participants, 14 were women, and 2 were men. Twelve of the managers had a background in care work, having previously worked as assistant nurses or in other caregiving roles. Seven participants held degrees in social work, with some overlap between those with care backgrounds and those with formal social work education. The participants’ experience as managers ranged from newly appointed to up to 28 years in a managerial role.

### Data collection

Semi-structured interviews were conducted between 7 February and 3 May 2023, lasting 52–93 min (average 69 min). All interviews were conducted face-to-face, except for one, which was held digitally via Zoom. The interviews explored managers’ experiences with temporary workers, focusing on their roles, the proportion of temporary staff, and the challenges and opportunities posed by different employment forms. Additionally, managers discussed their approaches to maintaining a good work environment in relation to staffing structures. The interview guide was developed for this study and used as a flexible framework for the semi-structured interviews (see Supplementary material 1). The interview guide included background questions, followed by in-depth questions on key topics such as staffing ratios, the impact of temporary employment on care quality, and strategies to address work environment challenges. All interviews were audio-recorded with consent and transcribed verbatim for analysis.

### Data analysis

The data were analysed using qualitative content analysis as described by Graneheim and Lundman [34] and further developed by Graneheim et al. [35]. This approach allows for an analysis that captures both manifest content, referring to what managers explicitly expressed, and latent content, which involves interpreting underlying meanings in the text. The analytical process followed an increasing level of abstraction. Meaning units were identified, condensed while preserving their essential meaning, and coded. These codes were then grouped into categories that reflect a low level of abstraction and remain close to the participants’ descriptions.

Two researchers were involved in the coding and interpretation process to strengthen the credibility of the analysis. Both had previous experience with qualitative interview studies and qualitative content analysis, with research expertise in eldercare and the work environment. AS, who conducted the interviews, carried out an initial analysis. TL then independently analysed all transcripts and listened to a selection of interview recordings, offering a second interpretation of the material.

These independent analyses were compared and discussed in detail, which helped to refine the categories and identify underlying themes. Although the category labels involve some interpretation, they largely represent the explicit content of the interviews. To further structure the findings, these categories were synthesised into broader themes, reflecting a higher level of abstraction [35]. The themes capture underlying perceptions about temporary staffing, its implications for management, and the challenges of balancing staffing needs with care quality. Additionally, all authors reviewed the final themes and categories to ensure consistency and confirmability.

## Results

**Fig. 2:**
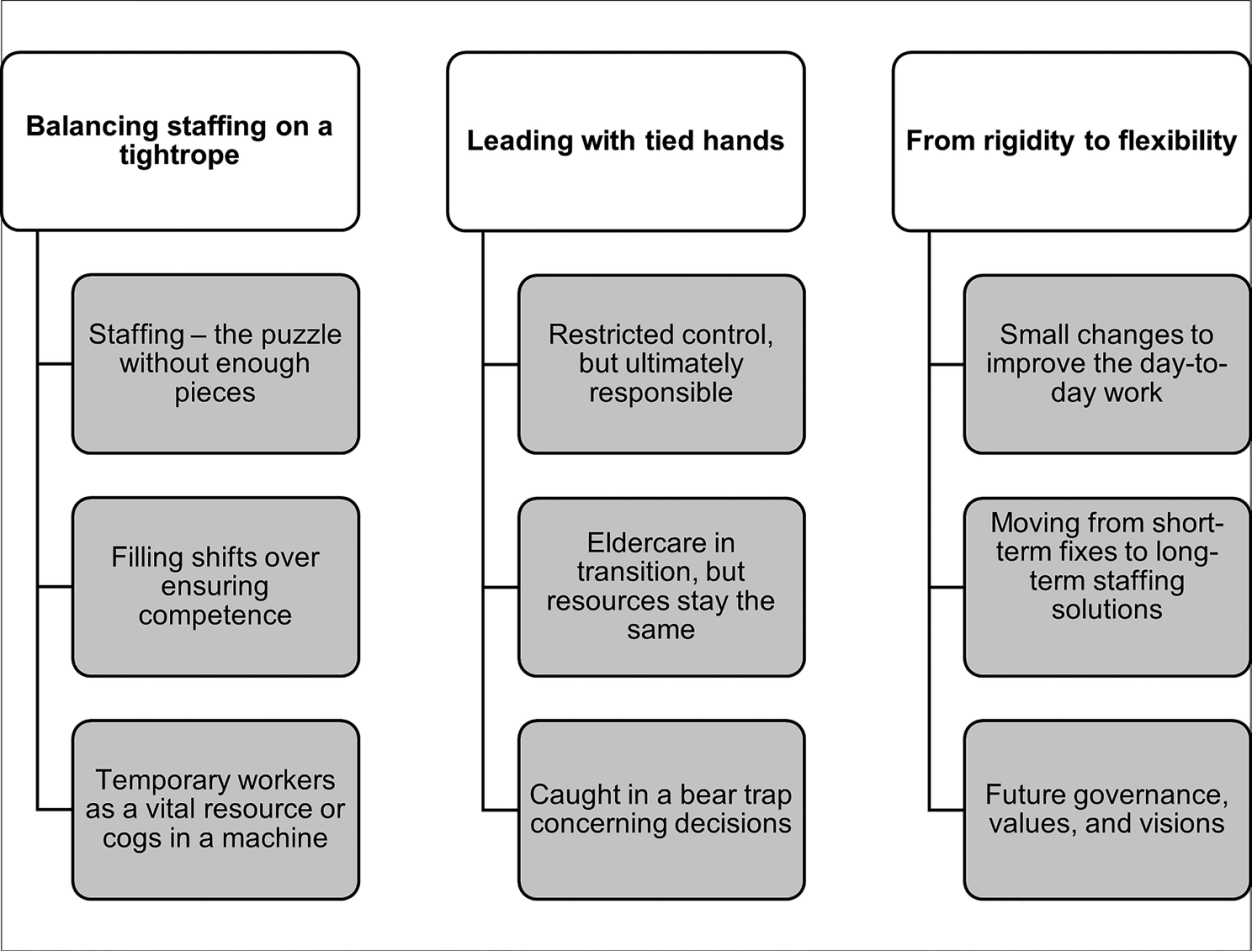
The themes at the top and the categories below each theme are based on interpretations of managers’ perceptions and experiences

Across all levels, the use of temporary staff is seen as both a necessary solution to staffing shortages and a challenge for ensuring continuity, quality, and a stable work environment, particularly when used extensively or without sufficient support. The findings highlight three key themes (Fig. 2 for an overview of themes and categories):

- *Balancing staffing on a tightrope* illustrates the benefits and challenges of managing temporary staff within a system that struggles to ensure continuity.
- *Leading with tied hands* captures managers’ experiences of unclear directives, systemic preconditions, and barriers that limit aspects of daily work.
- *From rigidity to flexibility* reflects managers’ perspectives on potential improvements, ranging from small-scale adjustments to fundamental structural reforms aimed at improving eldercare in general.

The themes are presented across managerial levels. When a finding was mainly expressed by managers at a particular level, this is specified in the text. Differences between managerial levels were most visible in how temporary workers were described as an organisational resource. Higher-level managers were more likely to link them to long-term workforce planning. In contrast, first-line managers focused more on the consequences for the daily work.

### Theme 1: Balancing staffing on a tightrope

**Fig. 3:**
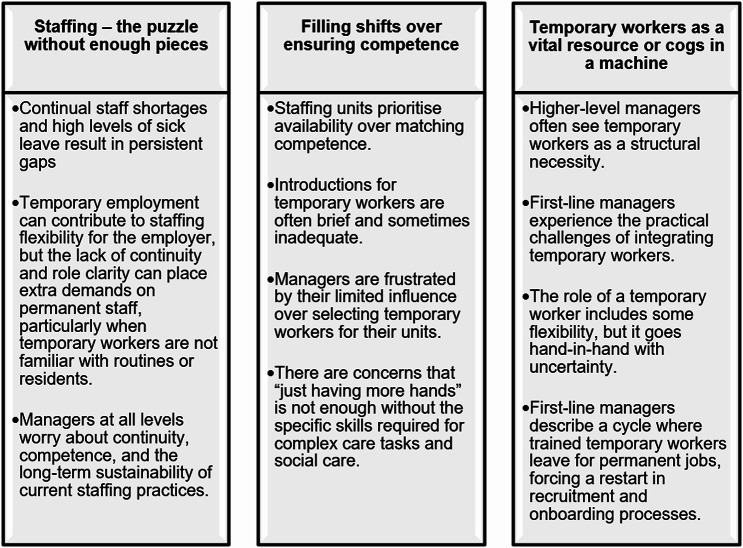
Overview of the key takeaways from theme 1

An overview of theme 1 is presented in Fig. 3.

#### Staffing – the puzzle without enough pieces

Several managers at different hierarchical levels emphasise the importance of stable units and long-term workforce planning to maintain continuity and quality of care. According to the managers, persistent staff shortages, high turnover, and budget limitations make this increasingly difficult. Despite efforts to retain employees, managers report that shifts are often left uncovered, leading to an ongoing reliance on temporary employment. Managers describe temporary workers’ flexibility as valuable for handling acute staffing needs. However, this unpredictability is a major concern for managers who worry about whether they will have enough staff, whether replacements will have relevant skills, or even if they will know certain tasks. Some first-line managers describe the situation as a “lottery”. Sometimes, it works out well when new temporary workers bring strong initiative, a good attitude, or a good fit with the team. Other times, temporary workers are new to both eldercare and the Swedish language, and first-line managers sometimes mention to higher-level managers how this can require time and support for them to become confident in their roles:

*There has been a genuine uncertainty. You never know if or whom you will get… Will I get any temporary workers? Will they be able to speak sufficient Swedish?* (IP1 higher-level management).

Managers describe workforce shortages as placing pressure on permanent staff. Managers at all levels note that excessive reliance on temporary employment can lead to fatigue among regular employees. They describe being expected to rely on temporary workers to cover staffing gaps, but in practice, staffing units often struggle to find them in time, even though they rely on them to fill urgent needs. Furthermore, sometimes, the temporary worker may be new and require support from permanent staff members. Consequently, permanent staff are sometimes required to work extra shifts, which may lead to exhaustion:

*We need to become as self-sufficient as possible. We do not have the budget to overstaff. Yet*,* we are constantly planning and relying on temporary workers we do not actually have. So we have to order permanent staff to work extra*,* and in the end*,* they cannot take it anymore.* (IP7 second-line manager).

Managers also describe high levels of sick leave as placing additional strain on staff, creating a vicious cycle of understaffing, increased workloads, and further sick leave. While temporary workers assist in filling gaps, they may not fully replace the experience or familiarity of permanent colleagues, which can increase the burden for the remaining staff:

*We do have high sick-leave numbers. Absolutely. Today*,* I have eight people on sick leave. And*,* of course*,* that costs. Because I need to bring in temporary workers for them as well. […] And then the work environment worsens for permanent staff. And then you end up in that situation… A negative spiral.* (IP12 first-line manager residential care).

Several first-line managers advocate for a more sustainable approach to breaking this cycle. Recruitment alone does not address the problem, and there is no universal quick fix. Managers emphasise the necessity of structured training and clear career pathways to strengthen the profession and reduce reliance on temporary employment. They report having begun utilising a new job role, service assistants, to manage non-care tasks, thereby allowing skilled personnel, such as assistant nurses, to concentrate on more complex responsibilities. Managers emphasise that without long-term workforce planning and clearer role divisions, reliance on temporary employment will continue to strain both the quality of care and employee well-being.

#### Filling shifts over ensuring competence

First-line managers describe how staffing practices in Swedish eldercare often prioritise filling shifts over ensuring long-term competence. The focus on addressing immediate needs has resulted in a system in which managers have limited influence over recruitment, and competence requirements frequently take a back seat to availability. One of the primary concerns is that temporary workers are assigned based on availability rather than suitability. They describe that the staffing unit sometimes focuses on simply filling vacancies, with little regard for whether the temporary worker has the necessary skills or ability to manage complex care situations. However, managers stressed that simply adding more “hands” is not enough if the person lacks training and competence:

*It is a complex job*,* really complex*,* and that is the problem. I am thinking of when temporary [workers] come in here. There is this idea that we just need more hands in care*,* more hands in eldercare. But the hands must belong to an educated body… If we take any hands*,* then we have a problem because not everyone can do this job. (*IP 15 first-line manager residential care).

The introduction process for new temporary workers is described by first-line managers as often minimal, especially in home care, with only online/digital videos, and then they receive only a few shifts before being expected to work independently. Given the complexity of eldercare, some managers argue that short introductions are insufficient, especially when many new hires lack prior experience: *“There is a lot they have to learn in a short time. Three shifts*,* and then they are expected to go out and work completely on their own.”* (IP 10 first-line manager home care).

Despite these challenges, managers find it difficult to remove unsuitable temporary workers when they are not a good fit for the role. There is no immediate way to reject someone who does not meet the necessary standards. Instead, first-line managers must go through a time-consuming process, holding repeated conversations with the employee and involving HR before any action can be taken. In the long run, managers fear that the continued focus on filling shifts rather than on ensuring competence will reduce the quality of care and working conditions for all in the sector.

#### Temporary workers as a vital resource or cogs in a machine

Managers describe temporary employment as a vital but imperfect resource for keeping services running during staffing shortages. Table 1 summarises the empirically identified priorities and perspectives across managerial levels. Second-line managers more often link temporary employment to efficiency and long-term workforce planning, whereas first-line managers focus more on the consequences for daily work, including strain on work groups and difficulties maintaining continuity. Managers also describe differences between care settings. In residential care homes, temporary workers usually have colleagues nearby; in home care, they more often work alone in older adults’ homes, making prior experience and familiarity with local routines especially important.

At higher organisational levels, temporary employment is primarily seen as a resource to fill staffing gaps. In contrast, first-line managers working directly with care delivery experience the effects of temporary workers firsthand. They describe how a lack of continuity and competence among temporary workers sometimes increases the workload for permanent staff. The reliance on temporary employment has also grown over the past decade. Furthermore, during the data collection period in spring 2023, one higher-level manager referred to January 2024 as a near-future target point in ongoing efforts to reduce reliance on temporary workers:

*If I look back ten years*,* I can say that the need for temporary workers has continuously increased. What is the likelihood that we will break that need by January 2024? There is no likelihood. (IP1* higher-level management).

While temporary employment is often viewed as primarily negative for overall continuity of care, some managers at different hierarchical levels described certain advantages. For the organisation, temporary workers enabled quick adjustments to fluctuating staffing needs and allowed permanent staff to attend training or take planned leave without major disruptions. Several higher-level managers also noted that, from the perspective of some temporary workers, temporary contracts could be attractive because they offered flexibility in when and how much they worked.

*There is a general view that being a temporary worker or using temporary employment is a bad thing. But I think that is an overly black-and-white view. It is not really like that. We will always need [that type of employment form] in this kind of service. There will always be unplanned absences*,* and there will also always be people who want to work that way. (IP 4* higher-level management).

**Table 1:** Priorities and perspectives on temporary employment across managerial levels

Role	Priorities	Perspectives on temporary employment
Higher-level management	Balancing political goals with strategic and operational planning. Prioritises organisational development, long-term workforce stability, and strategic staffing solutions. Open to overstaffing if possible.	Temporary workers are essential for managing unpredictability and seasonal variations. However, they are aware that skill mismatches can disrupt continuity of care. Managers hope to reduce reliance on temporary staffing.
Second-line managers	Ensuring a functional care system within budget limitations. Prioritises efficient staffing and scheduling to maintain stability and good care.	They are crucial resources for covering shortages but also pose risks to continuity and quality. Calls for a clearer strategy and better coordination.
First-line managers	They focus on resolving daily operational challenges, with particular attention to staff well-being. They emphasise continuity.	Temporary workers are needed but often lack adequate training. Managers want to reduce reliance on temporary workers to ensure continuity and quality.

According to most first- and second-line managers, temporary workers have fewer responsibilities than permanent workers. They describe not having to perform certain tasks, such as serving as a key contact for older adults or handling care plans. Some managers also note that this can make temporary work more appealing to those seeking flexibility rather than long-term commitment.

Furthermore, first-line managers emphasise how the recruitment of temporary workers has become a repetitive cycle. Many enter the sector through short-term contracts, gradually gaining experience and formal qualifications, only to leave for permanent positions elsewhere, forcing managers to restart the training process with new recruits.

Managers at all levels acknowledge that temporary workers are currently indispensable to the eldercare system. However, their value is closely linked to how they are integrated into daily work. When they return to the same units, receive sufficient introduction, and become familiar with local routines, they are described as an important resource. When they are used primarily to fill immediate gaps without adequate support, their contribution becomes more uncertain and can add pressure on already-strained work groups.

### Theme 2: Leading with tied hands

**Fig. 4:**
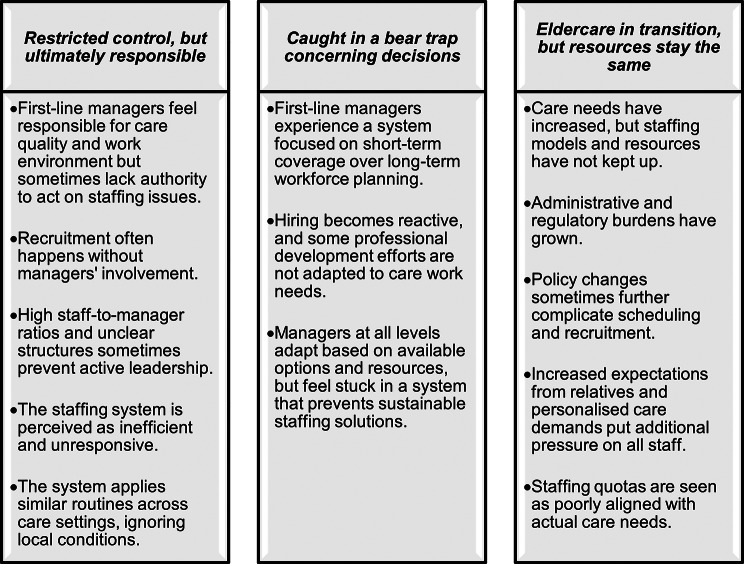
Overview of the key takeaways from theme 2

An overview of theme 2 is presented in Fig. 4.

#### Restricted control, but ultimately responsible

First-line managers express frustration over a system that lacks structure and clarity. Many feel they are expected to take responsibility without having the necessary tools. Second and first-line managers describe having little control over recruitment, as staffing decisions are often made centrally. Instead of selecting qualified candidates, they are assigned staff through automatic contract conversions, which limits their influence over who joins their units. Although managers are formally responsible for the quality of care and the work environment, they often lack the authority to make necessary staffing decisions or to address problems related to temporary workers. The limited influence over recruitment, together with high staff-to-manager ratios, makes it difficult for managers to provide the leadership and support they feel is needed. One first-line manager expressed the situation in a way that captures the frustration and resignation shared by many:

*Many times*,* you feel like your hands are tied. I am supposed to lead something with my hands tied. And just make the best of it… It is sad*,* but you have to learn to live with the feeling that you are not doing a good job. Otherwise*,* it will break you… I have 50 staff members who need quite a lot of coaching. That is simply impossible with the hours I have available… And since we do not do the recruiting ourselves*,* I get whoever I get. So I work with what I have and try to make the best of the situation.* (IP14, first-line manager residential care)

Several managers across levels describe clear differences between urban and rural settings, as well as between home care and residential care. For example, recruiting staff to rural areas is more difficult, public transport may be limited, and home care often requires a driving licence. Still, the system tends to treat all workplaces similarly, applying the same routines and expectations. This systemic approach makes it difficult for managers to adapt staffing and support to local conditions. First-line managers point out that each workplace has its own needs.

Consequently, managers often balance responsibility without authority, being expected to ensure quality and stability in a system where they may lack genuine control.

#### Caught in a bear trap concerning decisions

Managers at all levels, particularly first-line managers, describe a system in which short-term needs take precedence over long-term workforce planning. Limited budgets, strict policies, and bureaucracy hinder efforts to create stable units, invest in training, and retain staff. Some managers noted that focusing on one-year budgets fosters short-term thinking, making it difficult to invest in initiatives that would pay off later. They describe how spending may sometimes be necessary in the short term to save money or improve quality in the long term, but the system rarely allows for that. Although higher-level management encourages recruitment, first-line managers are reluctant due to financial uncertainty or structural limitations:

*I have not said no to hiring a single person. Hire them*,* I say. But then*,* further down in the organisation*,* we clearly do not do that. It might be a matter of: am I allowed to*,* do I dare to*,* can I*,* and what will the consequences be if I hire staff without the right qualifications?* (IP 1 higher-level management).

The higher-level managers also describe situations in which professional development initiatives do not align with the needs of care workers or the care profession because current structures are designed for other occupations or workplaces. Meanwhile, first-line managers express the feeling that they have little choice but to adapt to the system as it stands, making do with the staff available to them. Second and first-line managers feel trapped in a system that tightens its grip like a bear trap, leaving little room for strategic planning. They remain caught in a cycle of reactive decisions that wear down both themselves and staff.

#### Eldercare in transition, but resources stay the same

Managers across levels describe how eldercare has undergone major changes. However, available workers with sufficient time, resources, structures, and staffing levels have not kept pace. In home care, older adults remain in their own homes for as long as possible, while in residential care homes, residents now move in later in life and often require complex medical care.

*Eldercare today is not what eldercare was ten years ago. People are already ill when they arrive. They require more care. It is basically end-of-life care right from the start… But the resource allocation has not changed. Unfortunately.* (IP13 first-line manager residential care).

At the same time, administrative tasks have substantially increased. Managers at all levels highlight that new regulations, documentation requirements, and decentralised processes consume valuable time that could be devoted to direct care. Structural and political changes also introduce additional challenges. Policy reforms, new restrictions on split shifts, extended rest periods, and changes in working time regulations are expected to impact scheduling and staffing availability:

*Now the new EU directive is coming*,* stating that daily rest must be at least 11 h. […] And where are we supposed to find enough workers now*,* when we already struggle to recruit new staff*,* especially those with the right qualifications?* (IP2 higher-level management).

Another major shift is the increasing involvement of relatives in care decisions. First-line managers report growing expectations from families, sometimes exceeding available resources. These expectations place additional demands on staff. According to first-line managers, this increasing focus on individual preferences also signals a broader transformation in eldercare, where personalised care plays a more significant role, particularly in in-home care. The eldercare workforce is also undergoing demographic shifts. The gender distribution is changing, with more men entering the field, especially in home care: *“We now have about one-third of men in one of the teams.”* (IP8 first-line manager home care).

At the same time, workforce planning suffers from outdated staffing models. While care needs have increased, personnel quotas have remained unchanged or even decreased. *“When I started*,* the staffing ratio was 0.63. Today*,* it is 0.57. At the same time*,* we have more sick [residents] today and more demanding care needs. What is the thought process behind it?”* (IP16 first-line manager residential care). Some second- and first-line managers describe fixed staffing ratios and budget models as examples of organisational rigidity, as these do not sufficiently account for differences in care needs or for what is required to make schedules work in practice:

*We have the same staffing levels whether it is somatic care or dementia care. If we say we have this many residents*,* then we have a staff ratio of 0.57 during day and evening shifts. And that is what we have budgeted for. But to make the schedule work*,* maybe we have coverage for ten full-time positions*,* yet to actually fill the schedule*,* we need twelve people. (*IP 7 second-line manager).

Managers describe these figures as unclear and not well-aligned with actual care needs. They also point to a disconnect between satisfaction surveys, financial planning, and working conditions. Even when satisfaction survey results were positive, they rarely lead to concrete improvements. Instead, outcomes are sometimes acknowledged symbolically, such as with a celebratory cake, rather than used to guide development or influence resource allocation. Thus, as demands and regulations increase, eldercare continues to evolve, but resources and support have not kept pace.

### Theme 3: From rigidity to flexibility

**Fig. 5:**
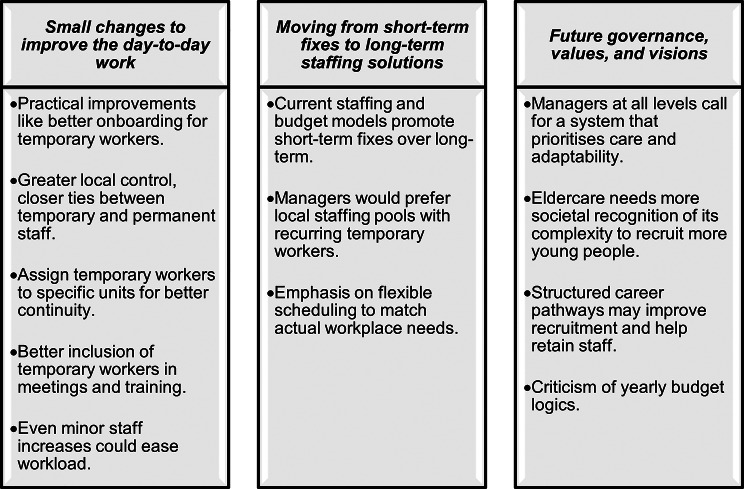
Overview of the key takeaways from theme 3

An overview and summary of theme 3 is presented in Fig. 5.

#### Small changes to improve the day-to-day work

Managers at all levels propose various improvements to make staffing more sustainable. Some solutions focus on structural changes, while others involve simple adjustments that could immediately ease staff burden and enhance the quality of care. A key theme is greater local control. Many managers at all levels feel that staffing decisions are too disconnected from daily work. They suggest closer ties between temporary workers and permanent staff, ensuring that temporary workers are not merely “extra hands” but are integrated into the unit.

*We are losing this part. Temporary workers are excluded from workplace meetings*,* workshops*,* and internal training sessions. If we want to make them more employable and competent*,* we need to include them more in the organisation.* (IP4 higher-level management).

Some argue that a dedicated pool of temporary workers, assigned to the same workplaces rather than being scattered across multiple sites, would increase both their competence and their job security. Improved onboarding processes for temporary workers is another priority. Some managers feel that new hires are too quickly dismissed as “not working out” when they could succeed with better training and support.

*It looks like we are giving them a chance*,* but we are not treating them the way we should. We judge them very quickly. […] How do we welcome them. How do we train them from the beginning? [We need to] Introduce them properly*,* use checklists*,* and teach them the most important things first.* (IP3 higher-level management).

Another challenge is that permanent staff often feel exhausted from constantly training new temporary workers. Ensuring a better match between temporary workers and workplaces, along with improved introductions, could help ease this burden. Furthermore, even minor additions to the workforce would make a significant difference.

*I would like to add one more staff member and a coordinator. That is two people. That is probably what it would take for things to work. That would eliminate a lot… A coordinator could pick up on things that come up during the day and relieve both managers and staff.* (IP15 first-line manager residential care).

While large-scale reforms may take time, these suggestions offer concrete ways to improve staffing stability and working conditions in the short term.

#### Moving from short-term fixes to long-term staffing solutions

Regarding long-term changes, first-line and some second-line managers express frustration with current staffing and budgetary models. Many argue that eldercare staffing should be planned proactively rather than relying on short-term fixes. The reliance on a reactive process creates instability and increases pressure on managers and permanent staff. They advocate greater autonomy in hiring and suggest establishing local staffing pools to improve continuity and ensure closer connections between temporary workers and the workplaces they serve.

*I would like to have my own pool of temporary workers closer to me. I would do the recruiting myself. I would be selective. […] I would take those who can work twice a month instead of being strict and saying you have to work at least 50% to be a temporary employee.* (IP12 first-line manager residential care).

Several managers highlight low staffing levels, which are based on financial calculations rather than actual care needs, and where shortcomings are often addressed with temporary fixes. Instead of over-relying on short-term contracts, managers suggest a higher baseline staffing level that allows for flexibility without sacrificing stability:

*I would overstaff to create stability and reduce the need for temporary workers… If you get that group*,* the stable one*,* then absenteeism naturally goes down. And I would rely on fewer temporary workers.* (IP12 first-line manager residential care home).

By giving managers greater control over hiring decisions and allowing them to create more workforce-adapted scheduling models, they believe eldercare could better balance flexibility and stability and, in turn, improve the overall quality of care.

#### Future governance, values, and visions

Managers at all levels advocate for different fundamental shifts in how eldercare is governed. They envision a system that prioritises relationships and individualised care over standardisation of care. One of the major challenges is attracting people to an increasingly demanding profession. Managers emphasise that eldercare work is often undervalued and misunderstood. Some find it frustrating when the profession is dismissed as unskilled or simple, pointing out the complexity of care work and how society fails to recognise its significance:

*To call this simple or unqualified work*,* to me*,* is very provoking. […] The pandemic shone a light on eldercare. Now*,* we need to invest in raising competence. But it is not that simple. There is a complexity that people do not really want to deal with*,* I feel.* (IP4 higher-level management).

Furthermore, managers note that fewer young people are interested in working in eldercare, and the profession is struggling with its public image.

*They [young people] see it as dirty work*,* and many have never had any contact with older people. That needs to change.* (IP8 first-line manager home care).

One proposed solution is to create clearer career pathways within eldercare, offering structured progression from entry-level positions to specialised roles. Furthermore, managers argue that structured exposure to eldercare at an early age, such as high school summer jobs, could help change perceptions and attract new generations to the sector. Ultimately, managers call for governance models that prioritise quality over regulation, enabling flexibility to meet the needs of older adults.

## Discussion

This study explored how managers at different levels in municipal eldercare perceive the challenges and opportunities related to temporary employment. The findings show that temporary workers are essential for managing staffing shortages, but heavy reliance on them creates difficulties in maintaining continuity and, at times, in maintaining care quality. Managers at all levels identified similar issues, but their priorities differed by role. First-line managers were more concerned with daily operational challenges, while higher-level managers focused on long-term workforce planning. A key finding is that staffing decisions in eldercare are strongly driven by short-term priorities, often prioritising quick shift coverage over matching workers’ skills to workplace needs. New temporary workers often receive insufficient training and onboarding, leaving permanent staff to compensate for their limited competence and familiarity with care routines. The staffing system often treats temporary workers as replaceable rather than as part of the team, which may reflect deeper structural problems. First-line managers are expected to take responsibility for quality and the work environment, but have limited influence over recruitment. Centralised hiring and tight budgets make long-term planning difficult. In addition, the same staffing routines are often applied across settings, whether in home care, residential care, or a unit in a rural or urban area. One way to understand the results is that the current system puts pressure on everyone involved: managers are expected to lead without enough influence, permanent staff are stretched thin, and temporary workers may lack support. This situation may undermine workforce stability, worsen the work environment, and affect the quality of care and service delivery.

### Managerial levels, governance, and long-term staffing

First-line and higher-level managers often have different priorities [25], and this study shows that such differences exist across multiple organisational levels. While all managers recognised the challenges associated with temporary employment, their perspectives reflected their positions: first-line managers emphasised daily staffing and work environment issues, whereas higher-level managers focused more on structural planning and long-term goals. This is consistent with previous research showing that managerial roles differ across levels. First-line managers typically address short-term demands, while middle managers are tasked with implementing policies from above, often with limited influence or support [29, 30]. When these roles are not aligned, implementation gaps can emerge [31].

In the present study, this misalignment was reinforced by short-term budgeting, unclear staffing ratios, and increased administrative demands. First-line managers described having to plan staffing within yearly budget cycles and staffing models that were experienced as poorly matched to actual care needs. This may reflect broader governance principles based on New Public Management, including cost-efficiency, strict budget control, and performance monitoring [26–28]. As a result, managers at different levels may work toward similar goals but under different time horizons and constraints. Connecting operational knowledge from first-line managers with strategic planning at higher levels may therefore be important for moving from short-term staffing fixes towards more stable and sustainable workforce planning in eldercare.

### Challenges in integrating temporary workers into the daily care units

Managers noted that new temporary workers in eldercare often receive limited training and have little familiarity with daily routines. This places extra pressure on permanent staff, especially in home care, where workers must act independently. Previous research has similarly linked temporary and precarious care work to insufficient training, limited supervision, job insecurity, and lower job satisfaction [5, 7, 14, 16, 22]. The findings also underline the importance of introductory practices, as good orientation to the work and the workplace has been associated with job satisfaction in long-term care [15].

Managers at different levels described temporary employment as a double-edged solution. It provides flexibility for both the organisation and, in some cases, the temporary worker, but also creates uncertainty for permanent staff, temporary workers, and managers. This aligns with previous findings from the same organisational context, in which temporary workers described both autonomy and vulnerability and reported lower job demands than permanent staff [8, 17]. In the present study, managers similarly reported that temporary workers often had fewer responsibilities. At the same time, permanent staff handled more complex tasks and supported less experienced colleagues. This suggests that temporary staffing may not simply redistribute work but may also shift responsibility and strain onto permanent staff. In line with previous long-term care research on staffing, quality, and organisational context, these findings suggest that staffing arrangements are closely linked to work environment and service delivery [13, 20, 21, 32]. Based on managers’ descriptions of the issue, our interpretation is that temporary employment may reinforce rather than relieve organisational pressure when support for temporary workers is insufficient, and task structures are unclear. This pressure concerns the organisation as a whole, but managers described it particularly regarding permanent staff, who often had to support temporary workers while maintaining continuity of daily care.

### Leadership under constrained staffing conditions

This study highlights several challenges first-line managers face in eldercare. In line with previous research, they often have limited influence over day-to-day decisions, including recruitment and staffing adjustments, despite being responsible for stability and quality [24, 25]. In the present study, this gap between responsibility and influence became particularly visible in relation to temporary employment. Managers were expected to ensure continuity, competence, and a stable work environment, but often had to work within centrally organised staffing systems, tight budgets, and policy decisions that limited their decision-making.

Managers also described how well-meaning political and regulatory decisions, such as stricter rules on shift schedules, mandatory rest periods, and limits on temporary employment, can have unintended consequences. While such policies aim to improve working conditions, they may reduce staffing flexibility and make it harder to adapt to changing care demands when resources are already stretched. Staffing decisions were therefore often experienced as reactive, with quick shift coverage taking precedence over matching workers’ skills with residents’ needs. This aligns with research describing gaps between available resources and rising demands in eldercare [4, 24], and with qualitative research portraying home care services as mainly reactive rather than proactive when capacity is stretched, and coordination is insufficient [36]. Research on distributed leadership similarly suggests that shared responsibility requires clear roles, dialogue-oriented relationships, and sufficient resources to function in practice [23]. Effective leadership in eldercare, therefore, requires more than individual managerial competence; it also depends on organisational conditions that enable managers to influence staffing decisions and shift from short-term fixes to more sustainable, long-term staffing.

### Methodological considerations

This study employed qualitative content analysis following Graneheim and Lundman [34], with a focus on credibility, dependability, and transferability. A strength of the study was the inclusion of managers at different hierarchical levels, which allowed for multiple perspectives on how temporary employment is handled in municipal eldercare. Using direct quotations helped ensure that the findings remained closely linked to the data.

While all qualitative research involves interpretation, the use of two independent analyses constitutes a strength of this study. Both AS and TL conducted separate analyses of the full dataset and compared interpretations in detail, which may improve the credibility and dependability of the findings [34]. Throughout the process, the authors continuously discussed the analysis. The categories remained close to the data, while themes reflected broader patterns based on managers’ descriptions, which aligns with Graneheim et al.’s [35] discussion of different abstraction levels in qualitative analysis.

A limitation is the relatively small sample of 16 managers. However, the interviews were long and detailed, and the sample included managers across several organisational levels and care settings. Another limitation is that the study focused on line managers and did not include HR specialists, staffing coordinators, or employees working in staffing units. These actors may also influence recruitment and workforce planning.

The Swedish context is both a strength and a limitation regarding transferability. A strength is that the study provides a detailed account of temporary employment in a publicly funded and municipally governed eldercare system, where managers are responsible for care quality but work within labour regulations, local budgets, and municipal staffing arrangements. The findings should therefore be interpreted in relation to this institutional context. At the same time, the challenges described in the study, such as reliance on temporary staffing and the difficulty of balancing short-term coverage with long-term workforce stability, may also be relevant to other welfare systems and health and social care contexts facing workforce shortages and extensive use of temporary or on-call staff, a challenge reported globally [1, 3, 32].

### Implications and future research

#### Practical implications

This study highlights several ways to improve the organisation of temporary employment in eldercare. Managers emphasise the need for stronger local control over staffing, including decentralised recruitment, to give first-line managers more influence over hiring and to make it easier to match workers’ skills with workplace needs [17, 23]. Creating staffing pools tied to specific units may support continuity and reduce the burden on permanent staff [8]. Structured onboarding with step-by-step learning and checklists can help temporary workers adapt more effectively while easing pressure on regular staff [3, 15]. Managers also stressed that temporary workers should, as far as possible, return to the same unit regularly to build competence and familiarity. Slightly increasing baseline staffing levels was seen as a way to avoid constant crisis recruitment. Finally, shifting from short-term budget logic to long-term financial planning may help eldercare move from reactive staffing towards more proactive workforce planning [4, 36]. In addition, managers call for broader discussions on how eldercare is governed, highlighting the need for stable funding and greater recognition of care work.

To address staffing challenges, it is important to understand why temporary employment is so widespread. The reasons may include sick leave, staff turnover, or broader structural conditions, and each may require a different response. In sectors like eldercare and education, where demanding work is combined with low pay and limited status, such conditions may make recruitment and retention particularly difficult.

#### Theoretical implications

This study offers three main theoretical points. First, temporary employment in eldercare does more than cover short-term staffing needs. It has become an integral part of how the system functions. While some flexibility is necessary, managers describe how current staffing models – shaped by political directives, regulations, centralised systems, and short-term budgets – limit their ability to plan proactively. This, in turn, influences how leadership is practised and what is possible in everyday management. Second, the findings point to a system that treats eldercare facilities, to some extent, uniformly. This makes it hard for managers to adapt staffing or support to what is needed on the ground in each home care or residential care home facility. Previous research shows that the organisation of work is important for the work environment in both home care and residential care homes [20, 21]. In this study, this was reflected in the ways temporary work differed across care settings: temporary workers in residential care homes often had colleagues nearby, whereas those in home care more often worked independently in older adults’ homes. Third, the study shows that the concept of span of control may need to be understood more broadly. It is not just about how many people a manager is responsible for, but also about the structure of the unit in terms of employment types, how often units change, how complex the work is, and how much support is available.

#### Suggestions for future research

In line with previous suggestions, more research is needed on sustainable staffing strategies in eldercare [3, 6, 32]. Future research could explore what drives the high use of temporary employment in eldercare, including sick leave, staff turnover, and broader structural conditions. Studies could examine whether improved working conditions reduce sick leave or whether clearer career paths improve retention. It may also be valuable to investigate differences in sick leave patterns between permanent and temporary staff, as well as how work organisation, job demands, control at work, and other work environment factors affect their well-being. Additionally, examining how managerial strategies impact the work environment and staff health could help identify more supportive leadership practices. One practical approach may be to evaluate structured onboarding for temporary workers and its effects on integration, job satisfaction, performance, and turnover.

## Conclusion

This study shows that temporary workers play a crucial role in municipal eldercare, but that reliance on temporary employment also creates challenges related to care continuity, workload, and organisational stability. Staffing practices often prioritise quick reactive solutions over proactive strategies that ensure workers have the right competence. As a result, temporary workers may be treated as interchangeable rather than as integrated team members, reflecting a systemic view of temporary staffing as a short-term fix rather than as part of building stable, high-quality care. Managers across all levels recognised the importance of temporary workers, but their perspectives differed by organisational role. First-line managers focused on the day-to-day consequences for staff and care quality, while higher-level managers emphasised long-term planning and resource distribution. To move towards more sustainable staffing, eldercare organisations need to rethink how temporary workers are integrated and supported. This includes structured onboarding, dedicated local staffing pools, and broader changes in how eldercare is governed. Recognising eldercare as a vital part of society means investing in long-term planning, valuing care work, and ensuring that leadership at all levels can work together toward common goals.

## Supplementary Information

Below is the link to the electronic supplementary material.

Supplementary Material 1

## Data Availability

The data supporting this study are not publicly available due to the General Data Protection Regulation (GDPR) and Swedish ethical guidelines.
