# Supplementary material for: Here today, gone tomorrow? Managerial perceptions of opportunities and challenges with temporary employment in eldercare
Source: BMC Health Serv Res. 2026 Jul 29;26:1031. doi: 10.1186/s12913-026-15246-y (PMC13422038; doi:10.1186/s12913-026-15246-y)
Supplement: Supplementary file 1 — Supplementary Material 1 [file 12913_2026_15246_MOESM1_ESM.docx]

**Supplementary material 1. Semi-structured interview guide with topics**

This interview guide was developed for the project and used as a framework for semi-structured interviews with managers. Before the interviews, the project was introduced and anchored both in writing and orally at all managerial levels.

# **Introduction at the interview:**

- Personal introduction
- Presentation of the purpose and aim of the study
- Description of why managerial perspectives are important
- Oral and written information about consent
- Review and signing of the consent form
- Opportunity for questions

# **Interview areas and questions**

## **Background information**

- Gender: ______
- Age: ______
- Education, including year of graduation: _____________________________
- Number of years worked as a manager: ______
- Number of years worked as a manager in the current position: ______

## **The participant’s role**

Please tell me about your current role and the organisation around you. What responsibilities do you have in your role?

**For example:**

- budget and personnel responsibility?
- responsibility for organisational development?
- responsibility for results or costs?
- number of employees?
- responsibility for the work environment?
- responsibility for recruitment?

## **Distribution and number of temporary workers**

Temporary employees in municipal eldercare are an important resource for keeping services running. We know that approximately one in five employees has a temporary employment contract.

- What is the distribution of different employment forms in the service or unit for which you are responsible?
- Is this distribution of employment forms appropriate, or would you prefer a different staffing mix?
- What possibilities do you have to influence the number of temporary employees?

## **Opportunities and challenges with different employment forms**

(Aspects such as finances, sustainability, leadership, employee engagement, and the work environment are addressed under each question)

Describe your experiences of:

- the opportunities associated with different employment forms in your service or unit.
- the challenges associated with different employment forms in your service or unit.
- (Follow-up question, if not covered elsewhere): How do you view the opportunities and challenges associated with different employment forms in relation to creating conditions for good care and running an effective service?
- How do you perceive your conditions, as a manager, for addressing the opportunities and challenges associated with different employment forms?

  How do you think employees with different employment forms would describe:
- the opportunities associated with different employment forms?
- the challenges associated with different employment forms?

## **Work environment management**

The work environment in a service is shaped by interactions among managers, permanent employees, and temporary employees.

- How do you work with work environment issues in relation to different employment forms?

## **Future suggestions**

- If you could choose freely, what would you like the staffing strategy to look like in your service or unit?
  (Follow-up question): What would be required for this staffing strategy to become possible?

Is there anything else you would like to add?
